# Identification of Close Relatives in the HUGO Pan-Asian SNP Database

**DOI:** 10.1371/journal.pone.0029502

**Published:** 2011-12-29

**Authors:** Xiong Yang, Shuhua Xu, The HUGO Pan-Asian SNP Consortium

**Affiliations:** Chinese Academy of Sciences Key Laboratory of Computational Biology, Chinese Academy of Sciences and Max Planck Society (CAS-MPG) Partner Institute for Computational Biology, Shanghai Institutes for Biological Sciences, Chinese Academy of Sciences, Shanghai, China; University of Cambridge, United Kingdom

## Abstract

The HUGO Pan-Asian SNP Consortium has recently released a genome-wide dataset, which consists of 1,719 DNA samples collected from 71 Asian populations. For studies of human population genetics such as genetic structure and migration history, this provided the most comprehensive large-scale survey of genetic variation to date in East and Southeast Asia. However, although considered in the analysis, close relatives were not clearly reported in the original paper. Here we performed a systematic analysis of genetic relationships among individuals from the Pan-Asian SNP (PASNP) database and identified 3 pairs of monozygotic twins or duplicate samples, 100 pairs of first-degree and 161 second-degree of relationships. Three standardized subsets with different levels of unrelated individuals were suggested here for future applications of the samples in most types of population-genetics studies (denoted by PASNP1716, PASNP1640 and PASNP1583 respectively) based on the relationships inferred in this study. In addition, we provided gender information for PASNP samples, which were not included in the original dataset, based on analysis of X chromosome data.

## Introduction

As the largest and most populous continent, Asia harbors substantial cultural and linguistic diversity, with its geographic structure of genetic variation remains enigmatic. The HUGO Pan-Asian SNP Consortium collected as many as 56,010 single nucleotide polymorphisms (SNPs) (54,794 on the 22 autosomes and 1,216 on X chromosome) from 1,719 DNA samples representing 71 Asian populations (Table S1) from China (including Taiwan), India, Indonesia, Japan, Malaysia, the Philippines, Singapore, South Korea and Thailand [1]. In the original data set, 45 Chinese (CHB, Han Chinese in Beijing), 44 Japanese (JPT, Japanese in Tokyo), 60 European-Americans (CEU, Utah residents with ancestry from northern and western Europe) and 60 Yoruba (YRI, Yoruba from Ibadan, Nigeria) from the International Haplotype Map Project (HapMap) were used as reference populations [2].

Accurate specification of relationships among individuals is critical for both medical and evolutionary genetic studies [3]. Unknown or misclassified relationships could lead to violation of assumptions of independence and decrease of power of the statistical inference. However, although considered in the original data analysis, the genetic relationships among individuals in the Pan-Asian SNP (PASNP) database were not clearly reported. We regarded it necessary to make clear panels without close relatives for future studies to refer to. Here, we performed a systematic analysis of genetic relationships among 1,719 (60 CEU, 60 YRI, 45 CHB and 44 JPT from the HapMap Project were not included in this study) individuals from PASNP. Based on the inferred relationships among individuals, three standardized subsets (denoted by PASNP1716, PASNP1640 and PASNP1583 respectively) of the original samples were suggested by avoiding monozygotic twins (MZ) pairs or duplicate samples, first-degree relationships and second-degree relationships for future applications in most types of population-genetics studies, following the procedures of the Human Genome Diversity Panel (HGDP-CEPH) [4] and the International Haplotype Map Project (HapMap) III [5].

## Materials and Methods

### Genotype Data

The 1,719 DNA samples from 71 Asian populations (Table S1) were genotyped with the Affymetrix Genechip Human Mapping 50 K Xba array and strict quality control was performed [1]. The data set is available in the Pan-Asian SNP database (PASNP) [6]. The genotype data released included 56,010 SNPs (54,794 on the 22 autosomes and 1,216 on X chromosome). One duplicate SNP (dbSNP ID, rs4028853) on X chromosome and 771 monomorphic SNPs (708 on the 22 autosomes and 63 on X chromosome) were removed. The data used for final analysis contained 55, 239 SNPs (54,086 on the 22 autosomes and 1,153 on X chromosome), only those SNPs with no missing genotypes in either of each pair were used for analysis. The samples were divided into 11 subsets according to the data sources when separate analysis was performed: Affymetrix, China, Indonesia, India, Japan, South Korea, Malaysia, the Philippines, Singapore, Thailand and Taiwan (Table S1).

### Gender Checking

Gender information was not available in the PASNP database [7], however, by constructing pedigree in this study, it should be very helpful in validating relationships and many other studies. Therefore, we employed the software PLINK (version1.07) [8] to check the gender of each individual in PASNP, and only the 1,153 SNPs on X chromosome were used in the analysis.

### Classical Multidimensional Scaling

To estimate genetic divergence among populations, we performed classical multidimensional scaling (MDS) with the software PLINK (version 1.07) [8] based on pairwise identical-by-state (IBS) distance using whole genome SNPs data. The value for parameter “*–mds-plot*” was 2. All possible pairs of the 1,719 individuals were analyzed with the 54,086 autosomal SNPs. Separate MDS analyses were also performed in the 11 subsets of the 1,719 samples (Table S1).

### Relative Pairs Analysis

To infer relationships among individuals, we employed the software KING (version 1.1.1) [9] which uses high-density genotype data and allows unknown population substructure. Only non-missing genotypes of the 54,086 autosomal SNPs in each pair were used in the analysis. All the 1,719 samples and the whole SNP data were analyzed to search for potential close relative pairs, and separate analyses were also performed in the 11 subsets. Substructures of populations were assumed and the parameters for KING were set as “–kinship –related” whose results were collected according to the manual of KING.

### Pairwise IBD Analysis

As for a pair of individuals, the proportion of the SNPs at which there were 0, 1, and 2 shared alleles identical-by-decent (IBD)—denoted by Z_0_, Z_1_, and Z_2_ respectively—was analyzed using the software package PLINK (version 1.07) [8] on each pair of the 1,719 individuals. Only non-missing genotypes of the 54,086 autosomal SNPs in each pair were used in the analysis.

## Results

### Gender Checking

The gender of an individual can be determined by inbreeding coefficient (homozygosity) estimation (denoted by F) based on X chromosome data. Following the recommendation of PLINK, we identified 742 individuals with F less than 0.2 as females, 919 individuals with F greater than 0.8 as males, and the remaining 58 individuals with F between 0.2 and 0.8 were treated as uncertain ones (Figure S1).

### Analysis of Individual Relationship and Population Stratification

**Figure 1:**
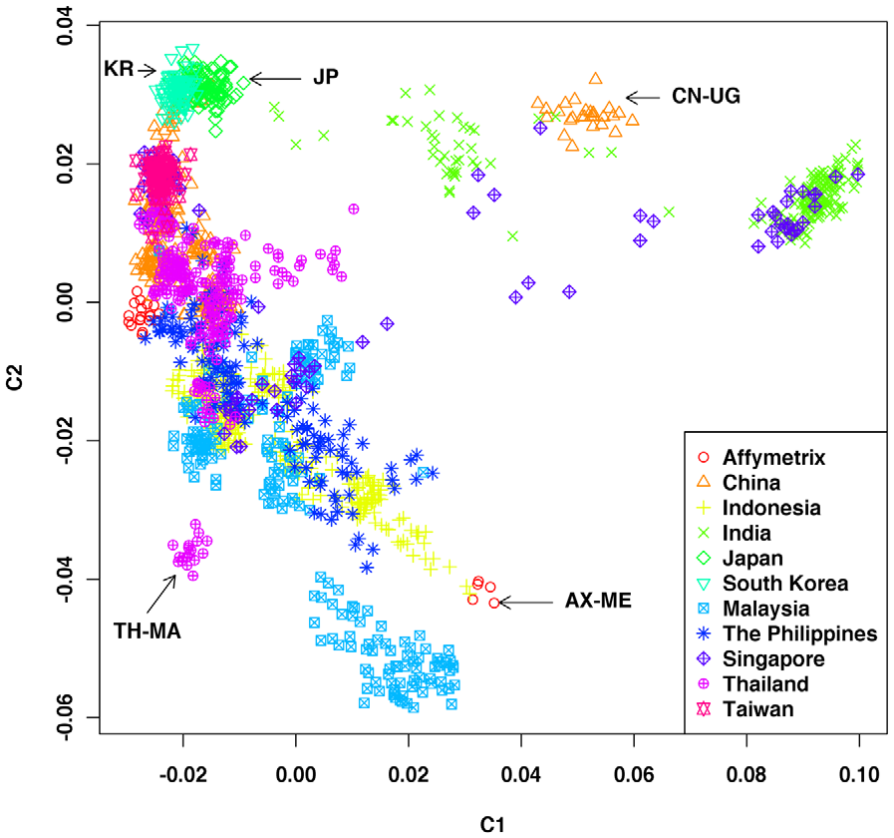
Classical Multidimensional Scaling (MDS) analysis performed on the 1,719 samples.

Individuals from the same population tend to cluster together based on genetic similarity. The MDS analysis was performed on the entire samples, with separated subsets revealing individual relationship and population stratification. Although population stratification was observed to some extent, considerable gene flow among populations was also observed in the MDS analysis on the full samples, as some individuals from different populations overlapped with others (Figure 1). The pattern is consistent with the structure inferred in a previous study [1]. The 18 individuals from Mlabri (TH-MA, Mlabri from Nan province, Thailand) clustered together within their population but did not overlap with the others.

Twenty-six Uyghur individuals (CN-UG, Uyghur from Hetian, Xinjiang, China) did not cluster with individuals from other populations from China but formed a distinct one slightly overlapping with the cluster of Indian individuals, which could reflect that the Uyghur is an admixed population with Western Eurasian ancestry [10], [11]. Similarly, five Melanesians individuals (AX-ME, Melanesians from Indo-Pacific) formed a distinct cluster and slightly overlapped with Indonesian populations. Individuals from India tend to disperse from each other, indicating a more significant genetic divergence among populations and individuals in India. In addition, the individuals from Japan and South Korea clustered tightly together and slightly overlapped with individuals from China, reflecting a close genetic relationship between Japanese and Korean populations, which was also observed in the previous study [1] (Figure 1).

Separate MDS analysis on the 11 disjoint subsets showed that individuals from India, Japan, South Korea, Singapore and Taiwan formed more dispersed clusters, while those from Affymetrix, China, Indonesia, Malaysia, the Philippines and Thailand each formed tight cluster(s) with a few exceptions (Figure S2, S3, S4, S5, S6, S7, S8, S9, S10, S11, S12). For example, in the MDS analysis of individuals from Affymetrix, they clustered into three groups: 5 individuals from AX-ME, 10 Ami individuals (AX-AM, Ami from Taiwan) and 9 Atayal individuals (AX-AT, Atayal from Taiwan) with an exception that one individual (AX-AT-013600-1-01) from AX-AT was between the latter two clusters (Figure S2). Similar results were also observed in the MDS analysis of other 10 subsets except South Korea and Taiwan (Figure S3, S4, S5, S6, S7, S8, S9, S10, S11, S12).

The individuals from one population usually clustered together except that one individual (IN-SP-000549-1-01) from an Indian population (IN-SP, Caucasoids from Haryana, India) was located in the dispersed cluster of another Indian population (IN-EL, Caucasoids from west Bengal, India), which might indicate mislabeling or a recent migrant (Figure S5). Similarly, one individual (SG-ML-000016-1-01) from SG-ML (Malay from Singapore) might also be mislabeled or an recent migrant (Figure S10).

Based on the results of MDS analyses on the whole samples and the 11 subsets, we concluded that genetic relationships among individuals in Asian populations were not that clear as self-reported or indicated by their geographical locations due to the considerable recent gene flow among populations.

### Relative Pairs Analysis

Based on the above analyses and observations, i.e. considerable gene flow among populations, separate analysis of close relative pairs within populations might lead to the missing of close relative pairs cross populations. In order to search for the possible close relative pairs among all the individuals, we employed the software KING to analyze the entire dataset and the 11 subsets. The results of whole data analysis and separate analysis were collected according to the manual of KING and were compared with each other. However, since false positive results could also occur as higher degree of relationships (first cousin, for example) might be inferred when the pairs of individuals were in fact not close relatives, only the relationships closer than or equal to first-cousin were collected when cross-country relationships occurred.

Pairs of individuals with estimated kinship coefficients greater than 0.354 were treated as MZ pairs (or duplicate samples); while those with kinship coefficient between 0.177 and 0.354 were treated as first-degree relationships; and those with kinship coefficient between 0.0884 and 0.177 were treated as second-degree relationships [9]. The proportion of a locus, at which a pair of individuals shared 0 allele IBD calculated by KING, was also used to classify the relationships between parent/offspring (PO) and full sibling (FS).

The kinship coefficient of a pair of individuals MY-TM-000022-1-01 and MY-TM-000025-1-01 from Proto-Malay (MY-TM, Proto-Malay from Malaysia) calculated was 0.177, which was just equal to the cutoff value of first- and second-degree relationships. However, to be conserved in constructing recommended subsets, we temporarily treated the pair of individuals as first-degree of relationships and more validation was performed in the next section. Similarly, the pair of individuals TH-MA-000124-1-01 and TH-MA-000128-1-01 from TH-MA with kinship coefficient as 0.0884 (equal to the second- and third-degree cutoff value) was temporarily treated as second-degree relationships. Furthermore, the inferred relationships collected from the whole data analysis and separate analysis were compared and turned out to be identical.

**Figure 2:**
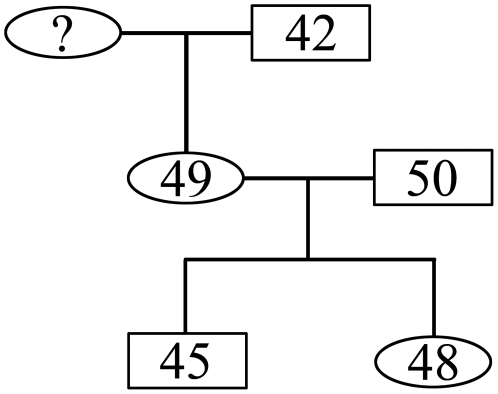
An example of pedigree constructed based on the inferred relationships. 42, MY-JH-000042-1-01; 45, MY-JH-000045-1-01; 48, MY-JH-000048-1-01; 49, MY-JH-000049-1-01; 50, MY-JH-000050-1- 01.

In some populations with many close relatives, especially those with large sample size, the number of relationships inferred could be very large and validating these putative relationships was complex. Therefore, pedigrees were constructed on the basis of the relationships inferred by KING to validate and classify PO pairs from FS pairs in the first-degree relationships inferred. For example, in the Negrito population (MY-JH, Negrito from Perak, Malaysia), both the individual MY-JH-000049-1-01 (female) and MY-JH-000050-1-01 (male) were inferred to have PO relationships with individual MY-JH-000045-1-01 (male), but the former two individuals were not inferred as close relatives. Individual MY-JH-000048-1-01 (female) was inferred to have PO relationships with MY-JH-000049-1-01 (female) and MY-JH-000050-1-01 (male); and to have FS relationship with MY-JH-000045-1-01 (male). In addition, the individual MY-JH-000042-1-01 (male) was inferred to have PO relationship with MY-JH-000049-1-01 (female), and to have second-relationships with MY-JH-000045-1-01 (male) and MY-JH-000048-1-01 (female). A pedigree was constructed as shown in Figure 2. Thus we found that the relationships inferred were consistent based on the pedigree. Similar analyses were performed on other relationships inferred and finally we obtained 3 pairs of MZ, 55 pairs of PO, 47 pairs of FS and 158 pairs of second-degree relationships. Further validation will be performed in the following section by pairwise IBD analysis.

### Pairwise IBD Analysis

Pairwise IBD analysis was used to assist validating the pairs of first-degree of relationships inferred by KING. Without genotyping errors and mutations, Z_0_ and Z_1_ of MZ or duplicate sample pairs are expected to be 0 and Z_2_ to be 1; Z_0_ and Z_2_ of PO pairs are expected to be 0 and Z_1_ to be 1; Z_0_ and Z_2_ of FS pairs are expected to be 0.25 and Z_1_ to be 0.5. Therefore, the proportion of IBD (denoted by PI_HAT, PI_HAT = P (IBD = 2)+0.5*P (IBD = 1)), which equals to or above 0.5, suggests that the pairs are related in first-degree or closer relationships. Those pairs with PH_HAT value greater than 0.44 (0.05 genotyping error rate and 0.01 mutation rate were assumed) were collected to search for potential close relatives. The correlation of PH_HAT value calculated by PLINK and that calculated by KING was tested, with the Pearson correlation coefficient to be 0.980 (Figure S13).

**Figure 3:**
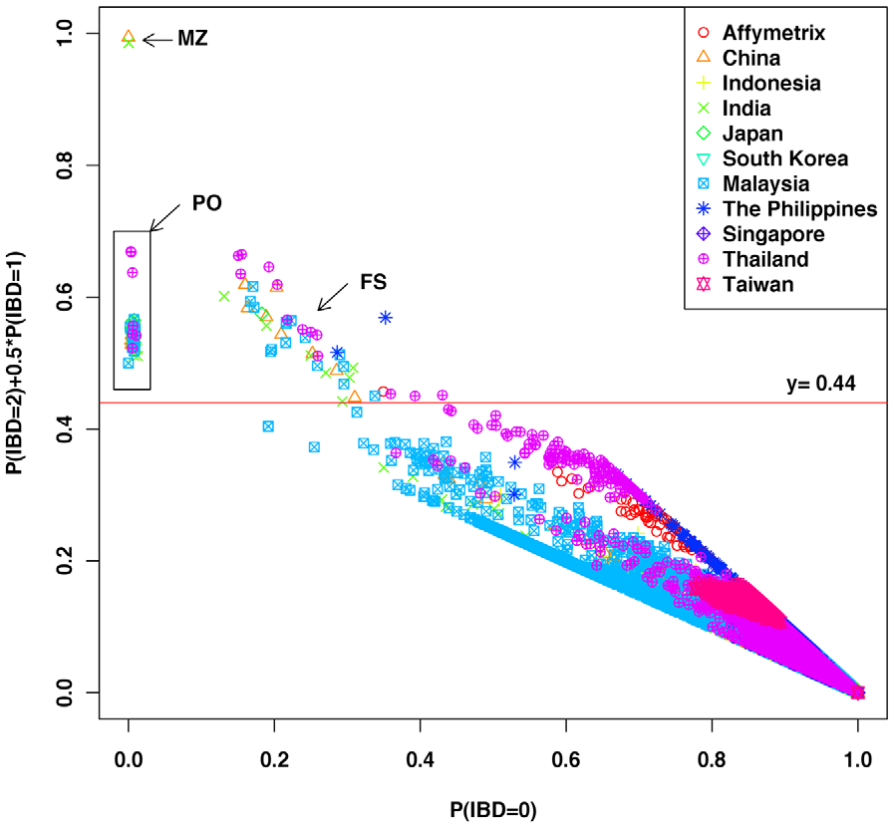
Pairwise IBD analysis of the 1,719 samples.

Three pairs of individuals with very high proportion of IBD (PI_HAT>0.99) suggested that they could be MZ or duplicate samples, and the MZ pairs inferred by pairwise IBD analysis were consistent with those inferred by KING, which were treated as “accurate” (Table S2). Fifty-five pairs (Figure 3) of individuals with relatively high proportion of IBD (PI_HAT>0.5) and Z_0_ near 0 (Z_0_<0.013) suggested that the 55 pairs could be PO pairs and were all found in the PO pairs inferred by KING. Therefore, we treated them as “accurate” (Table S3).

Forty-seven pairs (Figure 3) of individuals with relatively high proportion of IBD (PI_HAT>0.44) and relatively high Z_0_ (Z_0_>0.13) suggested that they could be FS pairs. Forty-three pairs of them were found in the FS pairs inferred by KING thus we treated them as “accurate” (Table S4). Two pairs (PI-MW-000022-1-01 and PI-MW-000029-1-01, PI-MW-000013-1-01 and PI-MW-000016-1-01) from Mamanwa (PI-MW, Mamanwa from Surigao del Norte, the Philippines) of the rest 4 were found in the second-degree relationships pairs inferred by KING and their IBDs were all greater than 0.49. To be conserved in constructing recommended subsets, we treated the two pairs as FS. However, one (TH-MA-000105-1-01 and TH-MA-000130-1-01) of the remaining 2 pairs from TH-MA with PI_HAT value near 0.45 was found in the second-degree relationships inferred by KING and the other pair (TH-MA-000103-1-01 and TH-MA-000122-2-01) was not found even in second-degree relationships inferred by KING. Since the Mlabri was a group of nomadic hunter-gatherers inhabiting the rural highlands in Thailand and were relatively isolated from other populations [12], [13], the inbreeding rate was expected to be relatively higher, so the genetic similarity between individuals would also be higher. Therefore we treated those two pairs as second-degree relationships rather than FS. At last, the 3 pairs (MY-KN-000008-1-01 and MY-KN-000019-1-01, MY-KN-000008-1-01 and MY-KN-000022-1-01, MY-KN-0000019-1-01 and MY-KN-000022-1-01) from Malay (MY-KN, Malay from Kelantan, Malaysia) and one (MY-TM-000022-1-01 and MY-TM-000025-1-01) from MY-TM of the 4 FS pairs inferred by KING but with PI_HAT value less than 0.41 were treated as second-degree relationships.

In summary, the final relationship pairs inferred by KING and validated by constructing pedigree and pairwise IBD analysis include: 3 pairs of MZ or duplicate samples, 11 parent/parent/offspring trios, 33 pairs of parent/offspring (PO) duos, 45 pairs of full sibling (FS), and 161 pairs of second-degree relationships (Table 1). Close relatives of first- and second-degree relationships were not observed in more than half (38) of the 71 populations, which suggested that the individuals from the 38 populations were “unrelated” according to the relationships we identified. The details of inferred relationships were summarized in Table S2, S3, S4, S5.

**Table 1:** Summary of inferred relationships.

POP	MZ	Trios	Duos	FS	2^nd^	POP	MZ	Trios	Duos	FS	2^nd^
AX-ME	0	0	0	0	1	MY-KN	0	0	0	0	37
CN-CC	0	0	0	2	0	MY-KS	0	0	1	4	11
CN-HM	1	0	4	2	1	MY-MN	0	0	1	0	5
CN-JN	0	0	0	0	3	MY-TM	0	4	4	7	25
CN-WA	0	2	0	5	2	PI-MA	0	0	0	0	1
ID-RA	0	0	1	0	1	PI-MW	0	0	0	2	0
ID-SO	0	0	0	0	1	TH-HM	0	0	0	0	1
IN-DR	1	0	0	0	0	TH-LW	0	0	0	2	2
IN-EL	0	0	2	2	4	TH-MA	0	0	3	5	5
IN-IL	0	0	1	1	2	TH-MO	0	0	1	0	0
IN-NL	0	0	0	1	1	TH-PL	0	0	0	0	2
IN-SP	0	0	1	1	0	TH-PP	0	0	0	0	1
IN-WI	0	0	1	0	1	TH-TK	0	0	0	0	1
IN-WL	1	0	0	1	0	TH-TL	0	0	0	2	0
JP-RK	0	3	2	1	0	TH-TN	0	0	2	1	3
MY-BD	0	0	2	2	12	TH-YA	0	0	1	1	1
MY-JH	0	2	5	2	25	*CROSS	0	0	1	1	12

POP, population ID; MZ, monozygotic twins; Trios, parent/parent/offspring; Duos, parent/offspring; 2^nd^, second-degree relationships; *CROSS, relationships cross populations.

### Construction of Recommended Subset PASNP1716, PASNP1640 and PASNP1583

Based on the relationships inferred above, we recommended, by avoiding MZ twins pairs (or duplicate samples), first-degree relationships and second-degree relationships, three standardized subsets (denoted by PASNP1716, PASNP1640 and PASNP1583) with different levels of unrelated individuals, and the following procedures were done on HGDP-CEPH panel [4] and HapMap III panel [5]. We constructed the standardized panels PASNP1716, PASNP1640 and PASNP1583 from the whole samples following the principles below:

1. Standardized subset PASNP1716 was constructed from the whole dataset, while PASNP1640 was constructed from PASNP1716, and PASNP1583 was further constructed from PASNP1640.
2. Adopted when discrepancy between different degrees of relationships occurred, lower degree of relationships were more reliable inferred by KING [9], and were treated as being more “accurate”.
3. Individuals were excluded in order to maximize the number of the remaining individuals in subsets recommended. Therefore the one with more occurrences in the same degree of relationships was preferentially excluded.
4. If two or more individuals with the same occurrence were in the same degree of relationships, the one with more missing data was preferentially excluded.

In summary, three individuals involved in MZ pairs were excluded from the whole dataset to construct standardized subset PASNP1716; seventy-six individuals involved in first-degree relationships were excluded from PASNP1716 to construct standardized subset PASNP1640; and 57 individuals involved in second-degree relationships were excluded from PASNP1640 to construct standardized subset PASNP1583. The individuals excluded were summarized in Table S6, S7, S8.

## Discussion

A comprehensive analysis was firstly performed to evaluate the genetic relatedness among samples from PASNP, the relationships inferred and the standardized subsets recommended, which will contribute to most of the future studies on medical and population genetics. In inference of close relative relationships, another software RELPAIR [14], [15] was also considered by using similar marker choosing strategy performed on the HapMap III samples [5] and 25 panels were analyzed. However, the number of markers RELPAIR allowed in analysis was no more than 10,000, and different marker choosing strategies might affect the relationships inferred, as observed in this study. For example, two individuals (MY-TM-000009-1-01and MY-TM-000017-1-01) from MY-TM were inferred as a FS pair (16 of the 25 panels) while sometimes inferred as a grandparent/grandchild (GG) pair (9 of the 25 panels, result not shown). In addition, among the 18 individuals from TH-MA, each pair of all the samples were inferred as FS pair, which was unlikely to be the real case. Therefore, we did not adopt the results inferred by RELPAIR but those by KING. Because KING relationship inference, using whole genome genotyping data, was not impacted by the linkage disequilibrium (LD) structure among adjacent SNPs and allowed unknown population substructure [9].

As Ani Manichaikul *et al* [9] pointed out in the original paper of software KING, with ∼5 k SNPs on the 22 autosome chromosomes, the performance of inferred relationships is reliable up to second-degree while third-degree relationships are reliable with 150 k SNPs. Therefore, it is proper to infer relationships up to second-degree based on our 50 k SNPs datasets. Furthermore, we also take one (CN-HM, Hmong from Southeast Guizhou Province, China) of the populations from China as an example to test the robustness of inferred relationships. Relationships among individuals in CN-HM were analyzed by randomly choosing 25%, 50% and 75% SNPs from the whole datasets, and the inferred relationships were compared (result not shown). The consistence of the relationships inferred by different densities of SNPs suggested that the 50 K SNPs used here were reasonable to infer relationships equal to or closer than second-degree relationships.

The existence of close relatives might affect the population structure and therefore leads to bias in assessing population stratification. To survey potential effects of close relatives on population structure, we compared the population structures with and without close relatives inferred by MDS analysis, taking the populations from China as an example. We found that the existence of close relatives did affect the results of population structure. However, the global population structure was not affected due to the fact that cross-population relationships did not exist (Figure S14). And the existence of close relatives within population only affected the local population structure (Figure S15). Therefore, we concluded that the population structure inferred by MDS analysis was reasonable despite the existence of close relatives within populations.

In pairwise IBD analysis, the relatively higher IBD value for the pairs of individuals from TH-MA could be resulted from inbreeding due to the small population size of this hunter-gatherer group [13]–[16]. And the large number of close relationships inferred in three populations (MY-JH, MY-KN and MY-TM) from Malaysia suggested considerable background relatedness in these populations. Special caution should be taken when relatedness among individuals matters.

## Supporting Information

Text S1 The list of the HUGO Pan-Asian SNP Consortium authors with their affiliations.
(PDF)

Figure S1 Check genders of the 1719 samples from PASNP. The individuals with homozygosity less than 0.2 were treated as females, greater than 0.8 were treated as males; and between 0.2 and 0.8 as uncertain (UN) ones.
(TIF)

Figure S2 MDS analysis of samples from Affymetrix.
(TIF)

Figure S3 MDS analysis of samples from China.
(TIF)

Figure S4 MDS analysis of samples from Indonesia.
(TIF)

Figure S5 MDS analysis of samples from India.
(TIF)

Figure S6 MDS analysis of samples from Japan.
(TIF)

Figure S7 MDS analysis of samples from South Korea.
(TIF)

Figure S8 MDS analysis of samples from Malaysia.
(TIF)

Figure S9 MDS analysis of samples from the Philippines.
(TIF)

Figure S10 MDS analysis of samples from Singapore.
(TIF)

Figure S11 MDS analysis of samples from Thailand.
(TIF)

Figure S12 MDS analysis of samples from Taiwan.
(TIF)

Figure S13 Comparison of IBD calculated by PLINK and that of KING. IBD = P(IBD = 2)+0.5*P(IBD = 1); PCC, Pearson correlation coefficient.
(TIF)

Figure S14 MDS plot of populations from China. (A) Population structure inferred by MDS analysis with close relatives. (B) Population structure inferred by MDS analysis without close relatives.
(TIF)

Figure S15 MDS plot of population CN-WA. (A) Population structure inferred by MDS analysis with close relatives. (B) Population structure inferred by MDS analysis without close relatives.
(TIF)

Table S1 Sample information of 71 Asian populations in Pan-Asian SNPs Database, and divided into 11 disjoint subsets when separate analysis needed.
(XLS)

Table S2 Three pairs of monozygotic twins or duplicate samples inferred. a, the proportion of two individuals shared alleles identity-by-decent (IBD) calculated by PLINK; b, the proportion of two individuals shared alleles IBD calculated by KING.
(XLS)

Table S3 Fifty-five pairs of parent/offspring pair inferred. a, the proportion of two individuals shared alleles identity-by-decent (IBD) calculated by PLINK; b, the proportion of two individuals shared alleles IBD calculated by KING.
(XLS)

Table S4 Forty-five pairs of full sibling pair inferred. a, the proportion of two individuals shared alleles identity-by-decent (IBD) calculated by PLINK; b, the proportion of two individuals shared alleles IBD calculated by KING.
(XLS)

Table S5 One hundred and sixty-one pairs of second-degree relationships inferred. a, the proportion of two individuals shared alleles identity-by-decent (IBD) calculated by PLINK; b, the proportion of two individuals shared alleles IBD calculated by KING.
(XLS)

Table S6 Three individuals excluded from whole samples to construct standardized subset PASNP1716.
(XLS)

Table S7 Seventy-six individuals excluded from PASNP1716 to construct standardized subset PASNP1640.
(XLS)

Table S8 Fifty-seven individuals excluded from PASNP1640 to construct standardized subset PASNP1583.
(XLS)
