# Supplementary material for: Identification of Close Relatives in the HUGO Pan-Asian SNP Database
Source: PLoS One. 2011 Dec 29;6(12):e29502. doi: 10.1371/journal.pone.0029502 (PMC3248454; doi:10.1371/journal.pone.0029502)
Supplement: Text S1 — The list of the HUGO Pan-Asian SNP Consortium authors with their affiliations. (PDF) [file pone.0029502.s001.pdf]

Text S1. The participants of the HUGO Pan-Asian SNP Consortium are arranged by surname alphabetically in the following;

Mahmood Ameen Abdulla,[1] Ikhlak Ahmed,[2] Anunchai Assawamakin,[3,4] Jong Bhak,[5] Samir K. Brahmachari,[2] Gayvelline C. Calacal,[6] Amit Chaurasia,[2] Chien-Hsiun Chen,[7] Jieming Chen,[8] Yuan-Tsong Chen,[7] Jiayou Chu,[9] Eva Maria C. Cutiongco-de la Paz,[10] Maria Corazon A. De Ungria,[6] Frederick C. Delfin,[6] Juli Edo,[1] Suthat Fuchareon,[3] Ho Ghang,[5] Takashi Gojobori,[11,12] Junsong Han,[13] Sheng-Feng Ho,[7] Boon Peng Hoh,[14] Wei Huang,[15] Hidetoshi Inoko,[16] Pankaj Jha,[2] Timothy A. Jinam,[1] Li Jin,[17,37] Jongsun Jung,[18] Daoroong Kangwanpong,[19] Jatupol Kampuansai,[19] Giulia C. Kennedy,[20,21] Preeti Khurana,[22] Hyung-Lae Kim,[18] Kwangjoong Kim,[18] Sangsoo Kim,[23] Woo-Yeon Kim,[5] Kuchan Kimm,[24] Ryosuke Kimura,[25] Tomohiro Koike,[11] Supasak Kulawonganunchai,[4] Vikrant Kumar,[8] Poh San Lai,[26,27] Jong-Young Lee,[18] Sunghoon Lee,[5] Edison T. Liu,[8] Partha P. Majumder,[28] Kiran Kumar Mandapati,[22] Sangkot Marzuki,[29] Wayne Mitchell,[30,31] Mitali Mukerji,[2] Kenji Naritomi,[32] Chumpol Ngamphiw,[4] Norio Niikawa,[39] Nao Nishida,[25] Bermseok Oh,[18] Sangho Oh,[5] Jun Ohashi,[25] Akira Oka,[16] Rick Ong,[8] Carmencita D. Padilla,[10] Prasit Palittapongarnpim,[33] Henry B. Perdigon,[6] Maude Elvira Phipps,[1,34] Eileen Png,[8] Yoshiyuki Sakaki,[35] Jazelyn M. Salvador,[6] Yuliana Sandraling,[29] Vinod Scaria,[2] Mark Seielstad,[8] Mohd Ros Sidek,[14] Amit Sinha,[2] Metawee Srikummool,[19] Herawati Sudoyo,[29] Sumio Sugano,[36] Helena Suryadi,[29] Yoshiyuki Suzuki,[11] Kristina A. Tabbada,[6] Adrian Tan,[8] Katsushi Tokunaga,[25] Sissades Tongsima,[4] Lilian P. Villamor,[6] Eric Wang,[20,21] Ying Wang,[15] Haifeng Wang,[15] Jer-Yuarn Wu,[7] Huasheng Xiao,[13] Shuhua Xu,[37] Jin Ok Yang,[5] Yin Yao Shugart,[38] Hyang-Sook Yoo,[5] Wentao Yuan,[15] Guoping Zhao,[15] Bin Alwi Zilfalil,[14] Indian Genome Variation Consortium[2]

[1]Department of Molecular Medicine, Faculty of Medicine, and the Department of Anthropology, Faculty of Arts and Social Sciences, University of Malaya, Kuala Lumpur, 50603, Malaysia. [2]Institute of Genomics and Integrative Biology, Council for Scientific and Industrial Research, Mall Road, Delhi 110007, India. [3]Mahidol University, Salaya Campus, 25/25 M. 3, Puttamonthon 4 Road, Puttamonthon, Nakornpathom 73170, Thailand. [4]Biostatistics and Informatics Laboratory, Genome Institute, National Center for Genetic Engineering and Biotechnology, Thailand Science Park, Pathumtani 12120, Thailand. [5]Korean BioInformation Center (KOBIC), Korea Research Institute of Bioscience and Biotechnology (KRIBB), 111 Gwahangno, Yuseong-gu, Deajeon 305-806, Korea. [6]DNA Analysis Laboratory, Natural Sciences Research Institute, University of the Philippines, Diliman, Quezon City 1101, Philippines. [7]Institute of Biomedical Sciences, Academia Sinica, 128 Sec 2 Academia Road Nangang, Taipei City 115, Taiwan. [8]Genome Institute of Singapore, 60 Biopolis Street 02-01, 138672, Singapore. [9]Institute of Medical Biology, Chinese Academy of Medical Science, Kunming, China. [10]Institute of Human Genetics, National Institutes of Health, University of the Philippines Manila, 625 Pedro Gil Street, Ermita Manila 1000, Philippines. [11]Center for Information Biology and DNA Data Bank of Japan, National Institute of Genetics, Research Organization of Information and Systems, 1111 Yata, Mishima, Shizuoka 411-8540, Japan. [12]Biomedicinal Information Research Center, National Institute of Advanced Industrial Science and Technology, 2-42 Aomi, Koto-ku, Tokyo 135-0064, Japan. [13]National Engineering Center for Biochip at Shanghai, 151 Li Bing Road, Shanghai 201203, China. [14]Human Genome Center, School of Medical Sciences, Universiti Sains Malaysia, 16150 Kubang Kerian, Kelantan, Malaysia. [15]MOST-Shanghai Laboratory of Disease and Health Genomics, Chinese National Human Genome Center Shanghai, 250 Bi Bo Road, Shanghai 201203, China. [16]Department of

Molecular Life Science Division of Molecular Medical Science and Molecular Medicine, Tokai University School of Medicine, 143 Shimokasuya, Isehara-A Kanagawa-Pref A259-1193, Japan. [17]State Key Laboratory of Genetic Engineering and MOE Key Laboratory of Contemporary Anthropology, School of Life Sciences, Fudan University, 220 Handan Road, Shanghai 200433, China. [18]Korea National Institute of Health, 194, Tongil-Lo, Eunpyung-Gu, Seoul, 122-701, Korea. [19]Department of Biology, Faculty of Science, Chiang Mai University, 239 Huay Kaew Road, Chiang Mai 50202, Thailand. [20]Genomics Collaborations, Affymetrix, 3420 Central Expressway, Santa Clara, CA 95051, USA. [21]Veracyte, 7000 Shoreline Court, Suite 250, South San Francisco, CA 94080, USA. [22]The Centre for Genomic Applications (an IGIB-IMM Collaboration), 254 Ground Floor, Phase III Okhla Industrial Estate, New Delhi 110020, India. [23]Soongsil University, Sangdo-5-dong 1-1, Dongjak-gu, Seoul 156-743, Korea. [24]Eulji University College of Medicine, 143-5 Yong-du-dong Jung-gu, Dae-jeon City 301-832, Korea. [25]Department of Human Genetics, Graduate School of Medicine, University of Tokyo, 7-3-1 Hongo, Bunkyo-ku, Tokyo 113-0033, Japan. [26]Department of Paediatrics, Yong Loo Lin School of Medicine, National University of Singapore, National University Hospital, 5 Lower Kent Ridge Road, 119074, Singapore. [27]Population Genetics Lab, Defence Medical and Environmental Research Institute, DSO National Laboratories, 27 Medical Drive, 117510, Singapore. [28]Indian Statistical Institute (Kolkata) 203 Barrackpore Trunk Road, Kolkata 700108, India. [29]Eijkman Institute for Molecular Biology, Jl. Diponegoro 69, Jakarta 10430, Indonesia. [30]Informatics Experimental Therapeutic Centre, 31 Biopolis Way, 03-01 Nanos, 138669, Singapore. [31]Division of Information Sciences, School of Computer Engineering, Nanyang Technological University, 50 Nanyang Avenue, 639798, Singapore. [32]Department of Medical Genetics, University of the Ryukyus Faculty of Medicine, Nishihara, 207 Uehara, Okinawa 903-

0215, Japan. [33]National Science and Technology Development Agency, 111 Thailand Science Park, Pathumtani 12120, Thailand. [34]Monash University (Sunway Campus), Jalan Lagoon Selatan, 46150 Bandar Sunway, Selangor, Malaysia. [35]RIKEN Genomic Sciences Center, W502, 1-7-22 Suehiro-cho, Tsurumi-ku, Yokohama 230-0045, Japan. [36]Laboratory of Functional Genomics, Department of Medical Genome Sciences Graduate School of Frontier Sciences, University of Tokyo (Shirokanedai Laboratory), 4-6-1 Shirokanedai, Minato-ku, Tokyo 108-8639, Japan. [37]Chinese Academy of Sciences-Max Planck Society Partner Institute for Computational Biology, Shanghai Institutes of Biological Sciences, Chinese Academy of Sciences, 320 Yueyang Rd., Shanghai 200031, China. [38]Genomic Research Branch, National Institute of Mental Health, National Institutes of Health, 6001 Executive Boulevard, Bethesda, MD 20892 USA. [39]Research Institute of Personalized Health Sciences, Health Sciences University of Hokkaido, Tobetsu 061-0293, Japan.
