# Supplementary figures and images for: Identification of Close Relatives in the HUGO Pan-Asian SNP Database
Source: PLoS One. 2011 Dec 29;6(12):e29502. doi: 10.1371/journal.pone.0029502 (PMC3248454; doi:10.1371/journal.pone.0029502)

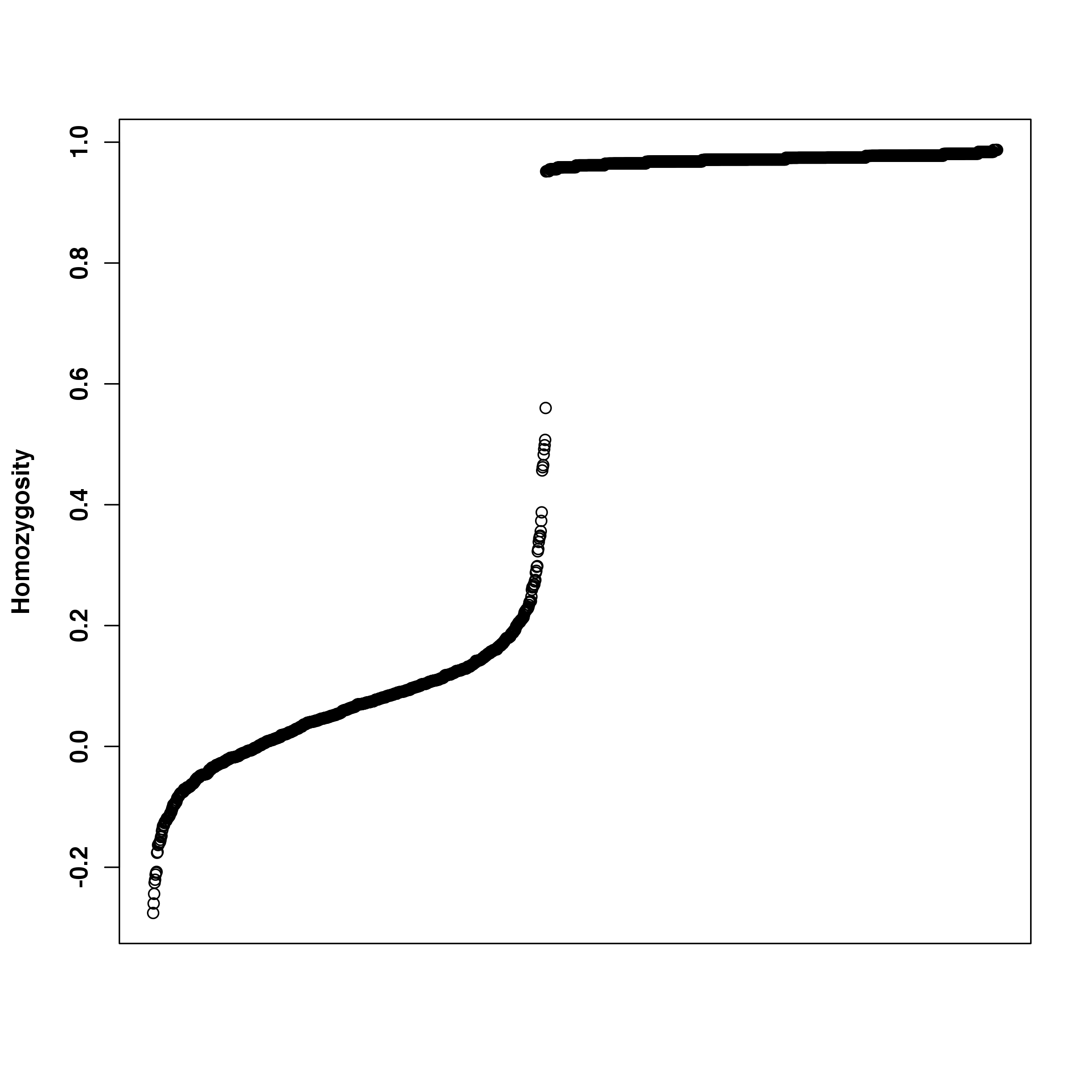

Supplement: Figure S1 — Check genders of the 1719 samples from PASNP. The individuals with homozygosity less than 0.2 were treated as females, greater than 0.8 were treated as males; and between 0.2 and 0.8 as uncertain (UN) ones. (TIF) [file pone.0029502.s002.tif]

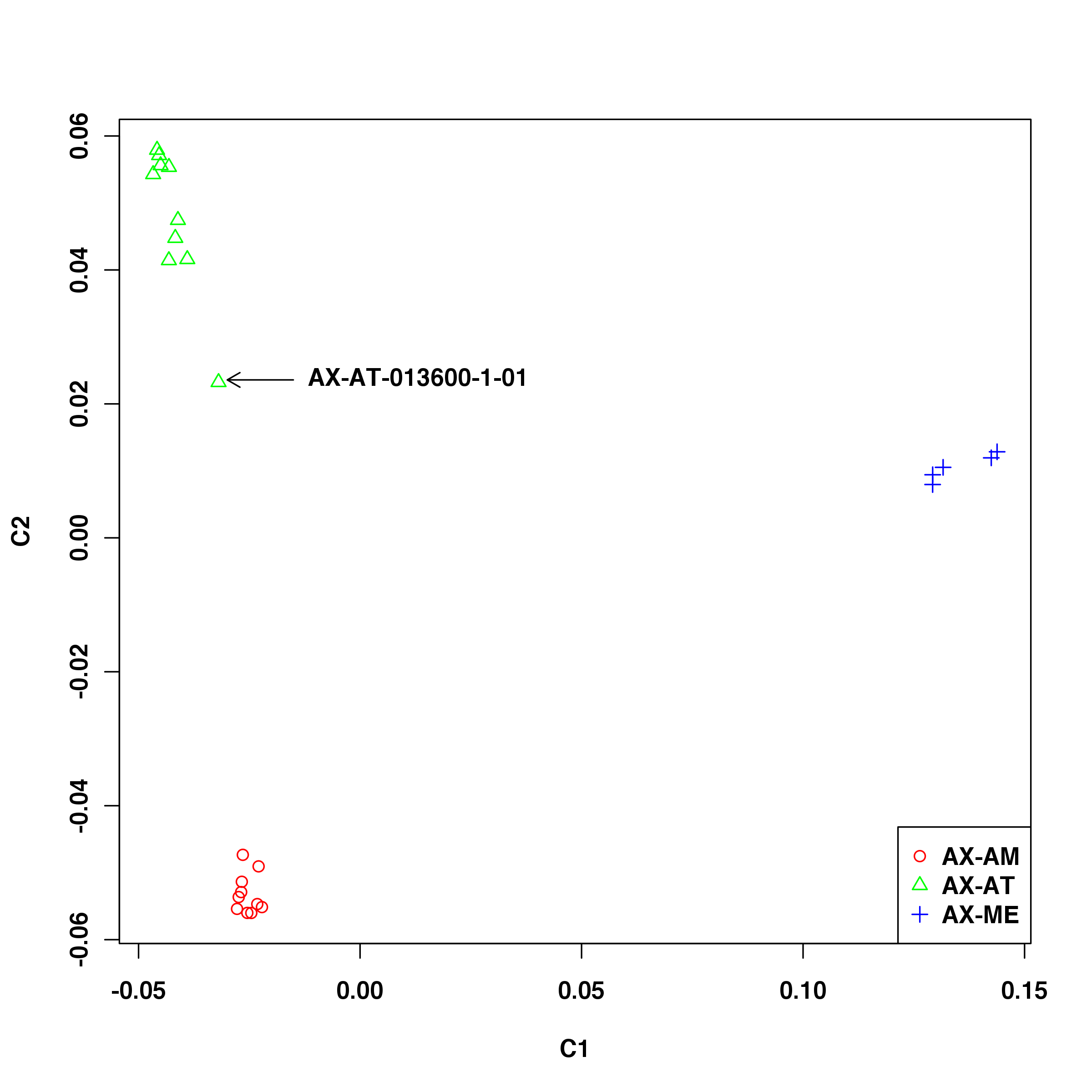

Supplement: Figure S2 — MDS analysis of samples from Affymetrix. (TIF) [file pone.0029502.s003.tif]

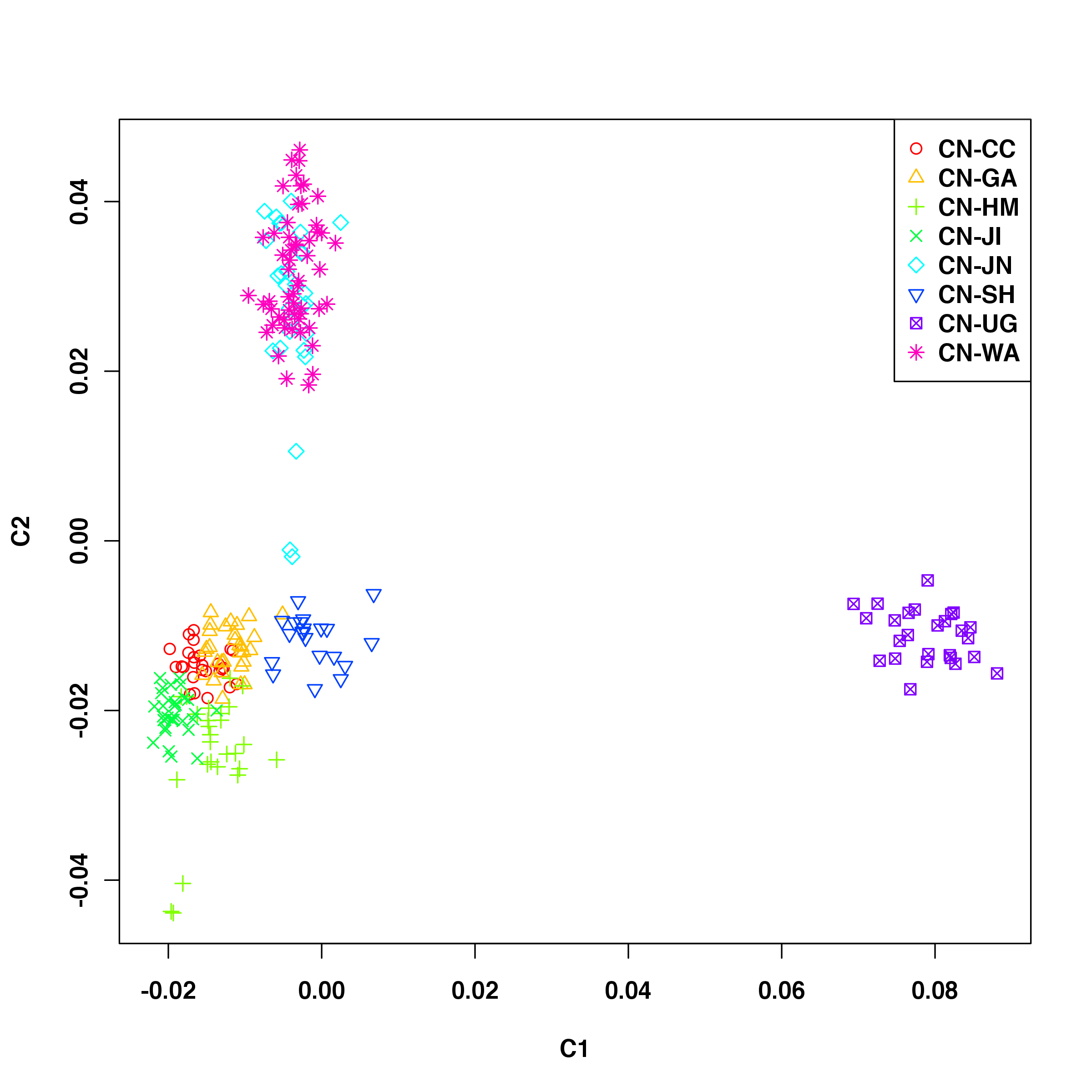

Supplement: Figure S3 — MDS analysis of samples from China. (TIF) [file pone.0029502.s004.tif]

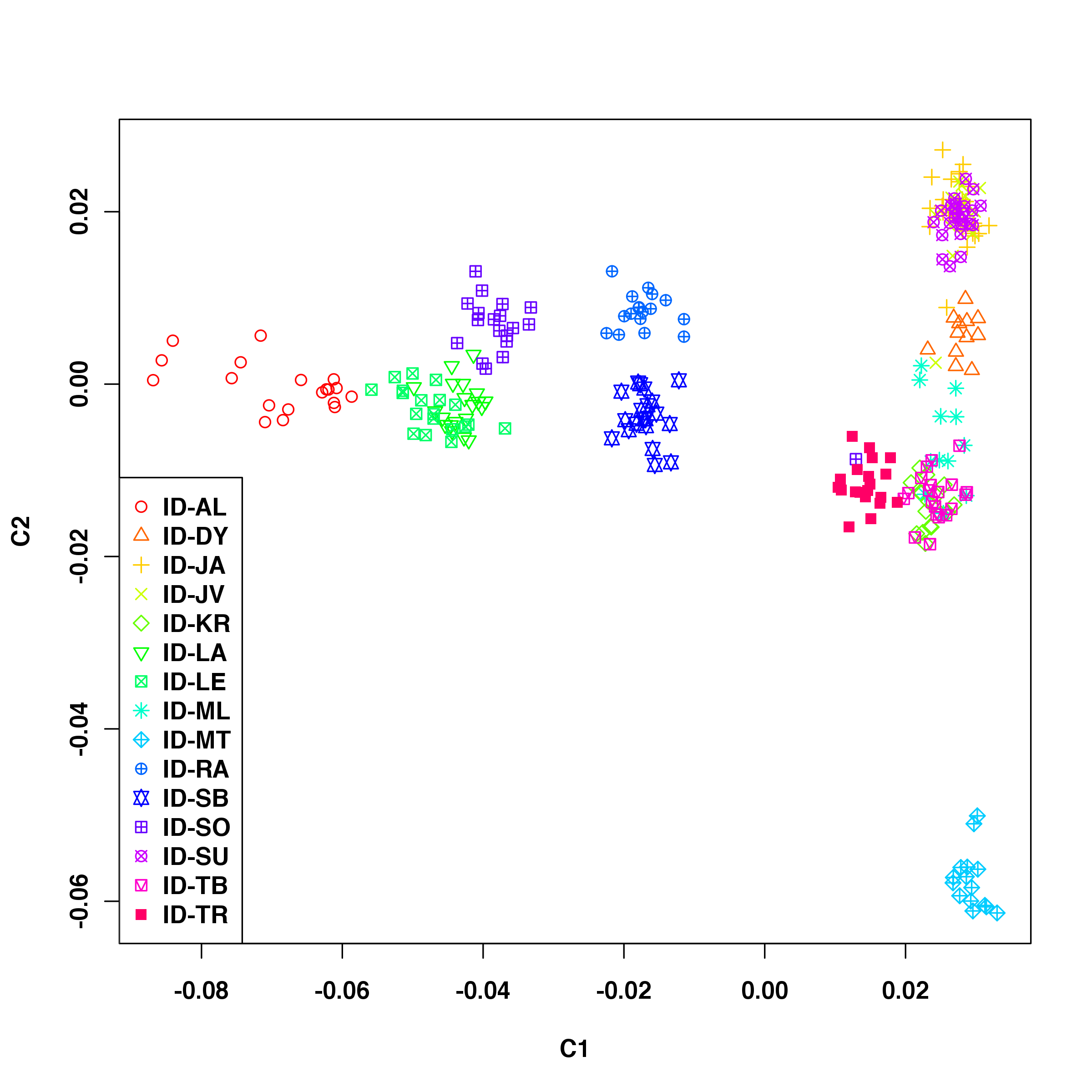

Supplement: Figure S4 — MDS analysis of samples from Indonesia. (TIF) [file pone.0029502.s005.tif]

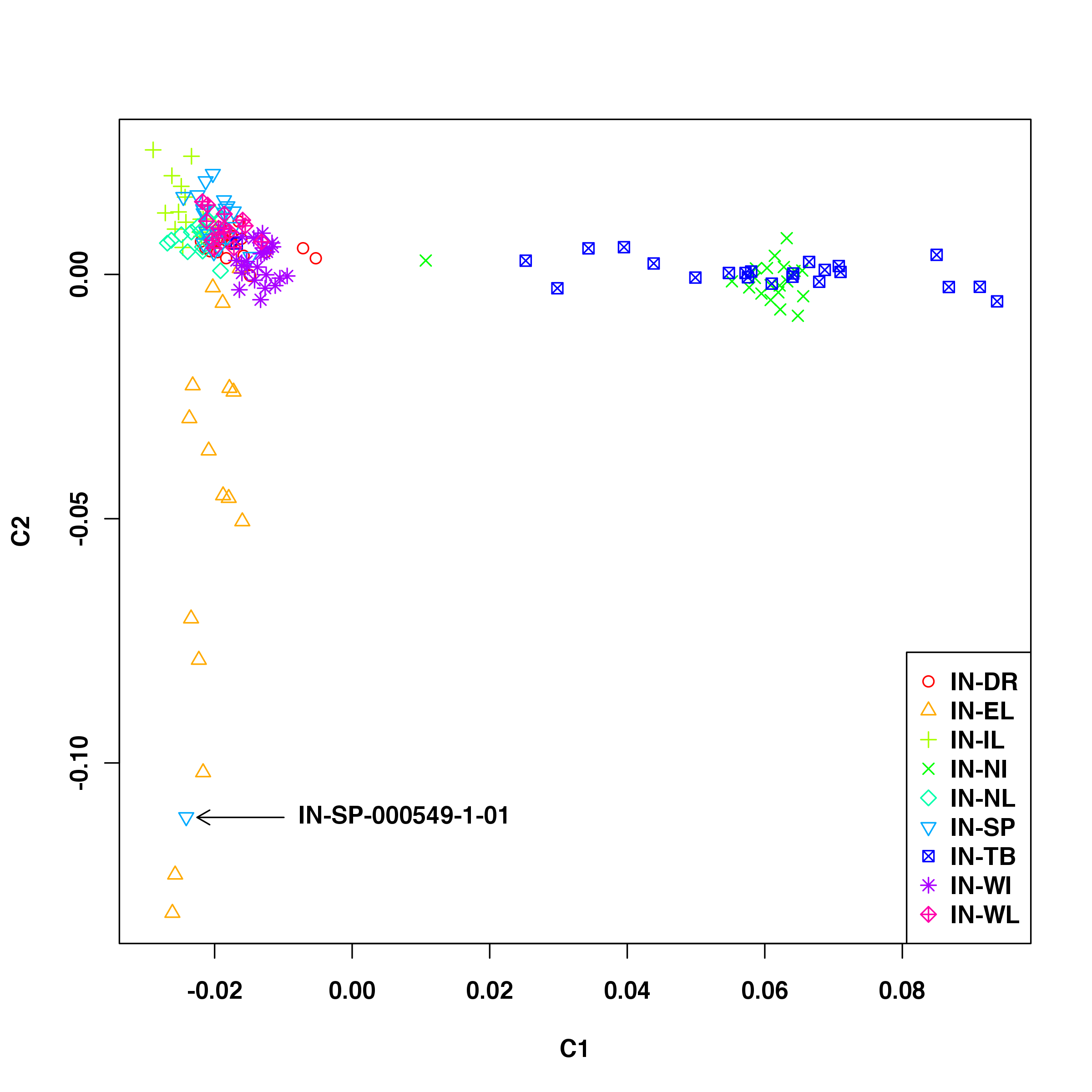

Supplement: Figure S5 — MDS analysis of samples from India. (TIF) [file pone.0029502.s006.tif]

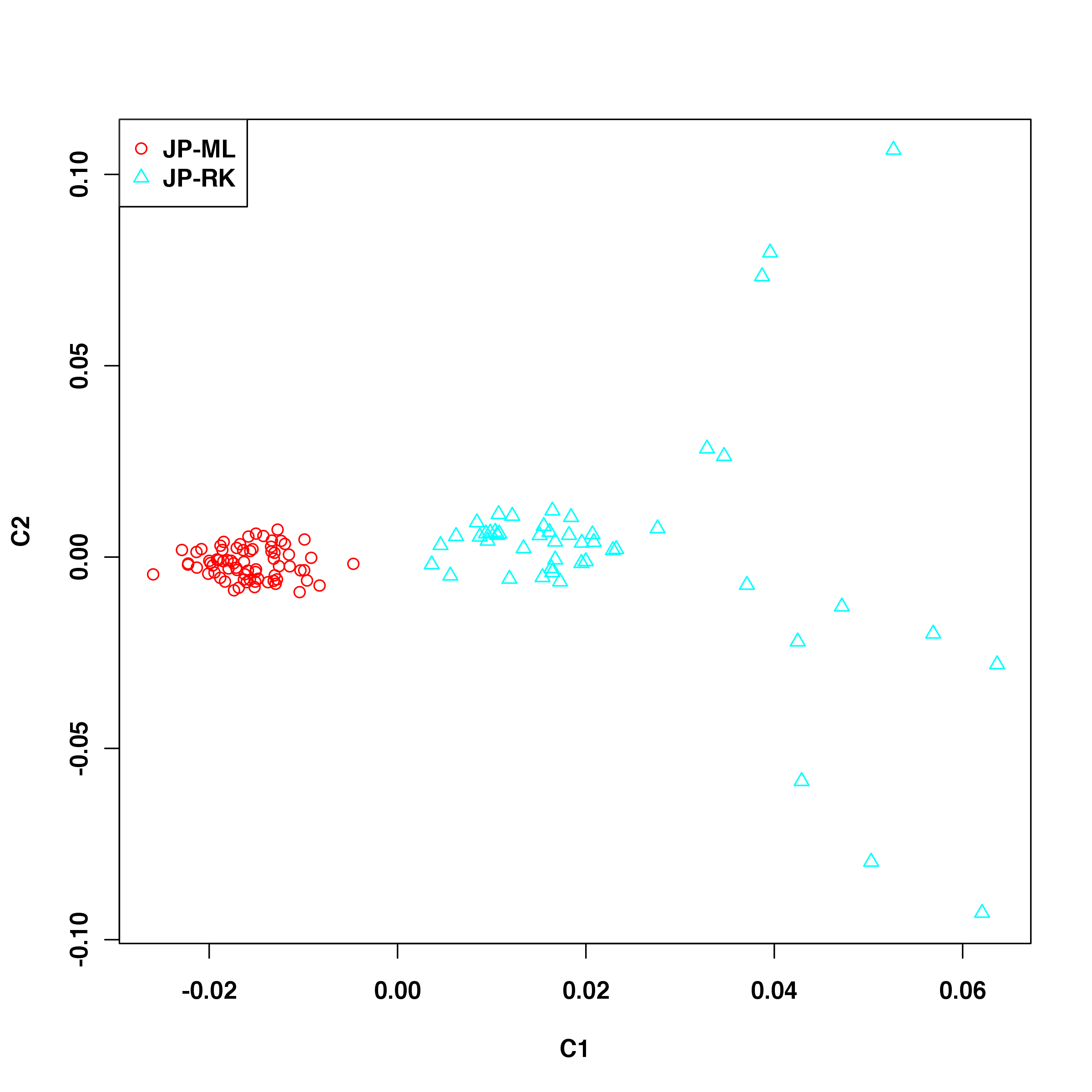

Supplement: Figure S6 — MDS analysis of samples from Japan. (TIF) [file pone.0029502.s007.tif]

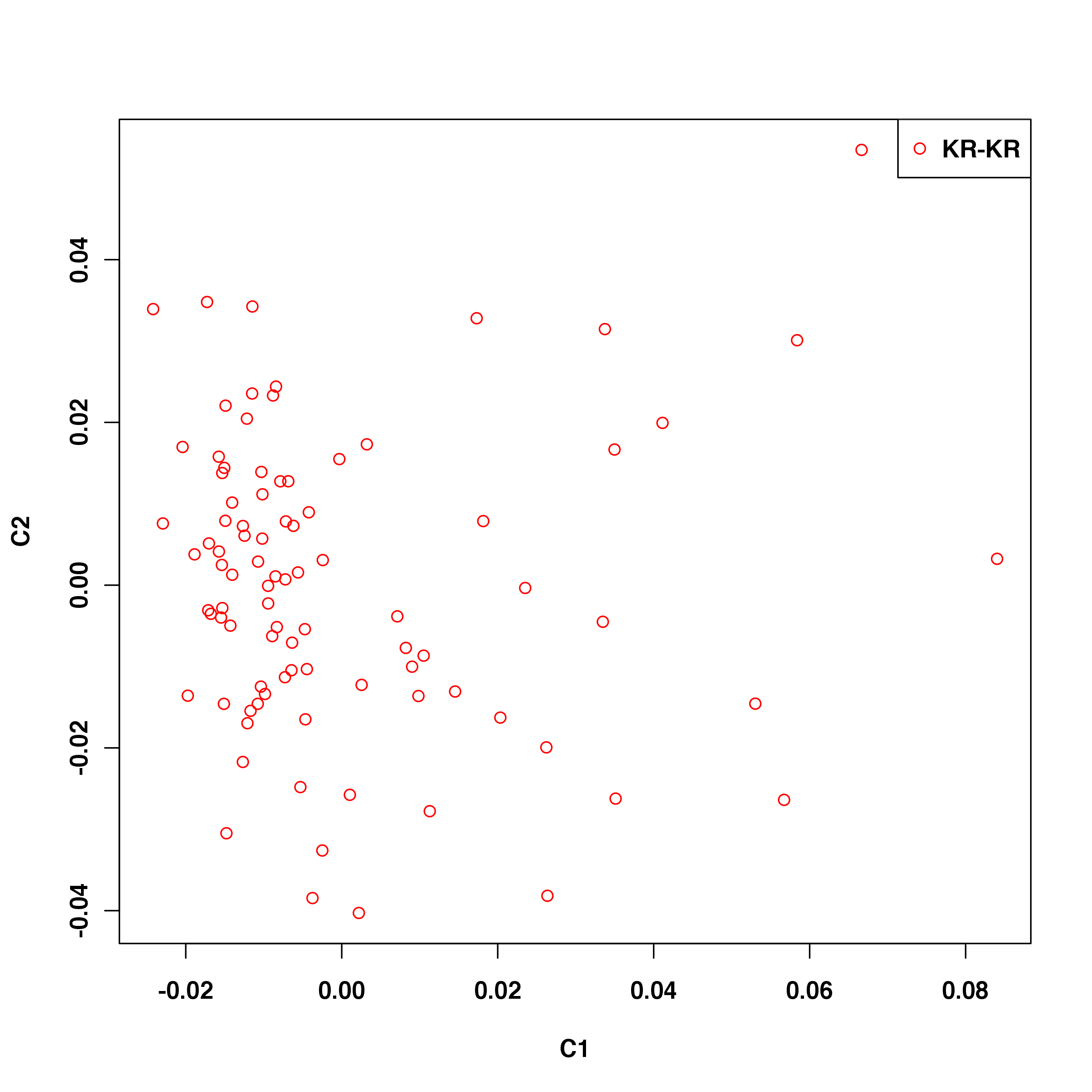

Supplement: Figure S7 — MDS analysis of samples from South Korea. (TIF) [file pone.0029502.s008.tif]

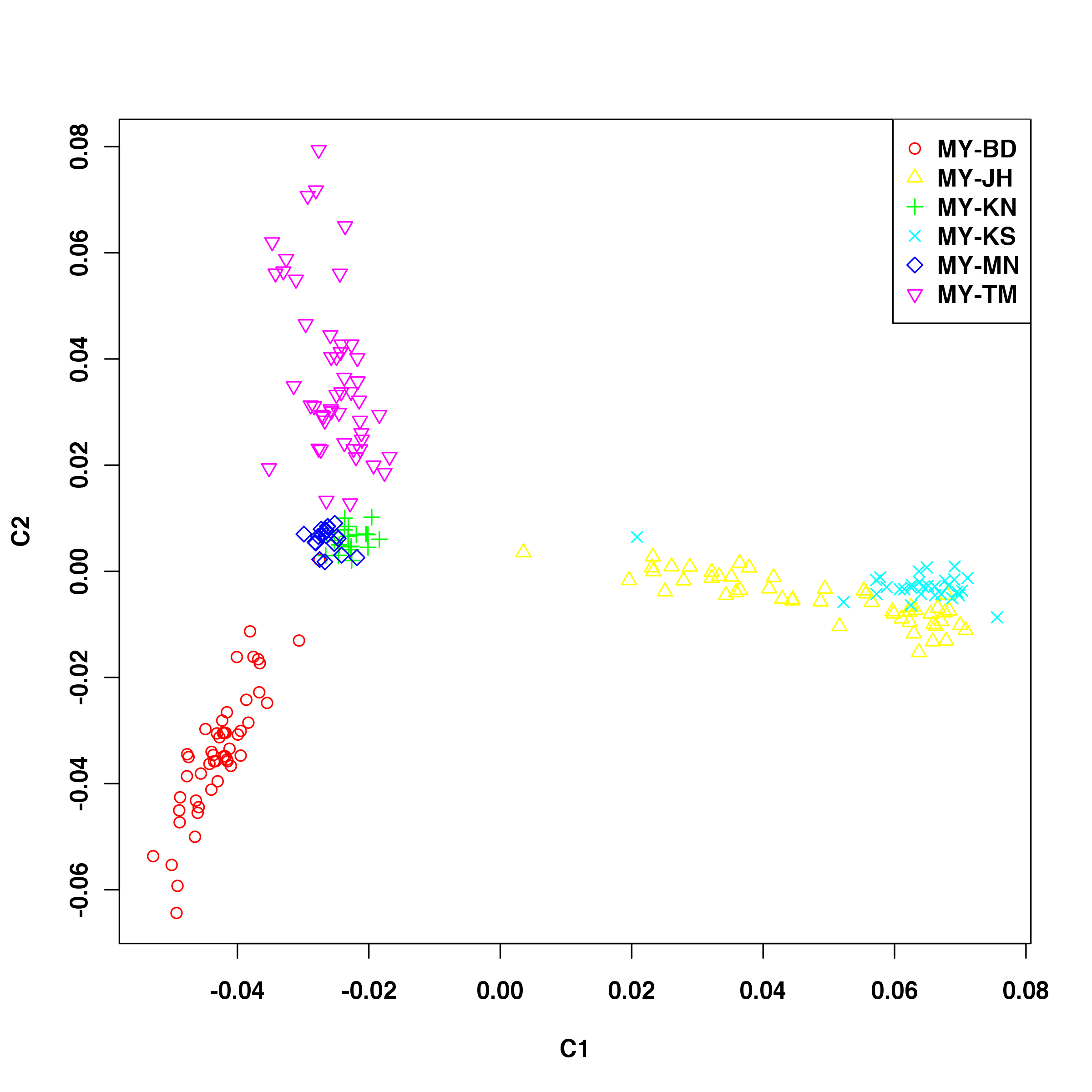

Supplement: Figure S8 — MDS analysis of samples from Malaysia. (TIF) [file pone.0029502.s009.tif]

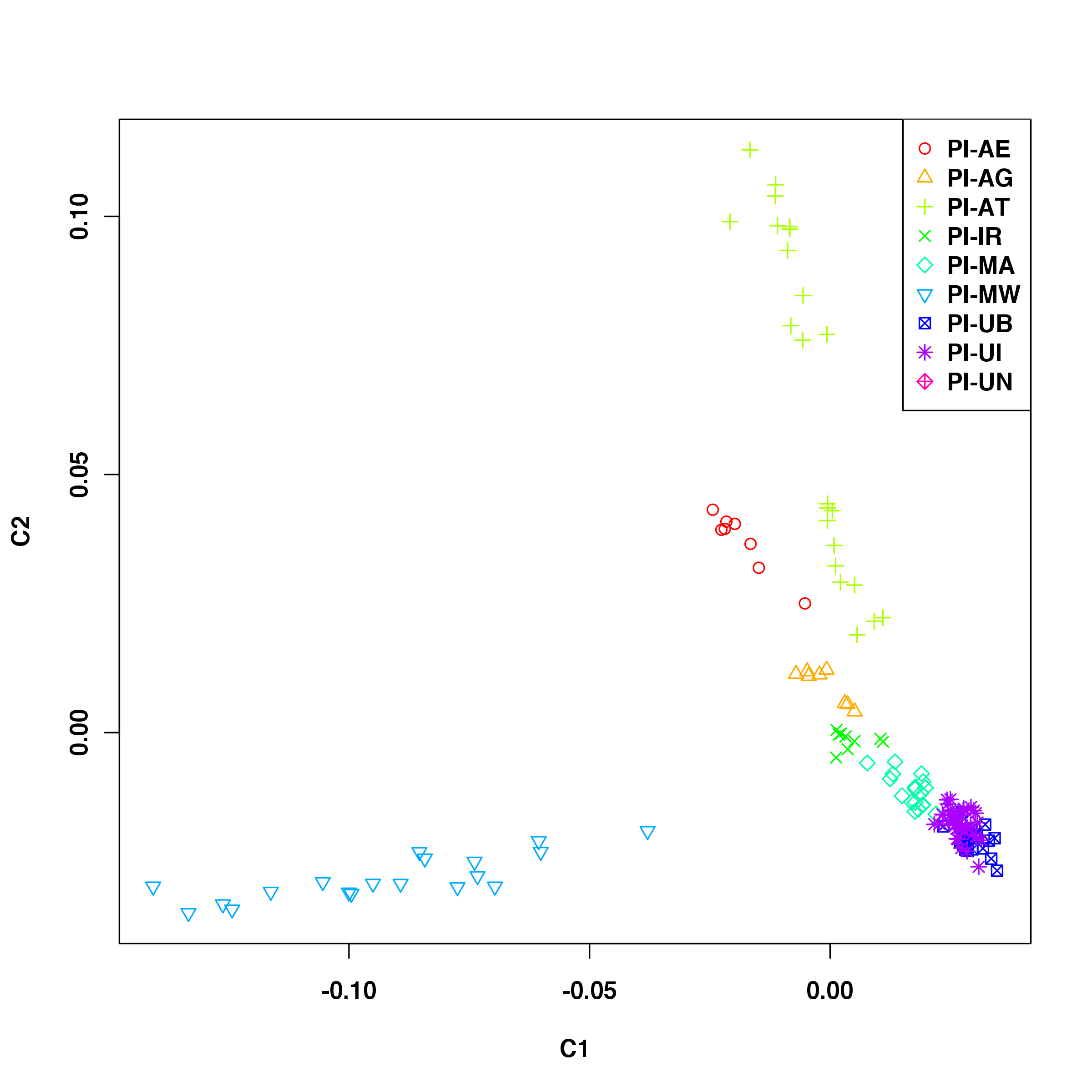

Supplement: Figure S9 — MDS analysis of samples from the Philippines. (TIF) [file pone.0029502.s010.tif]

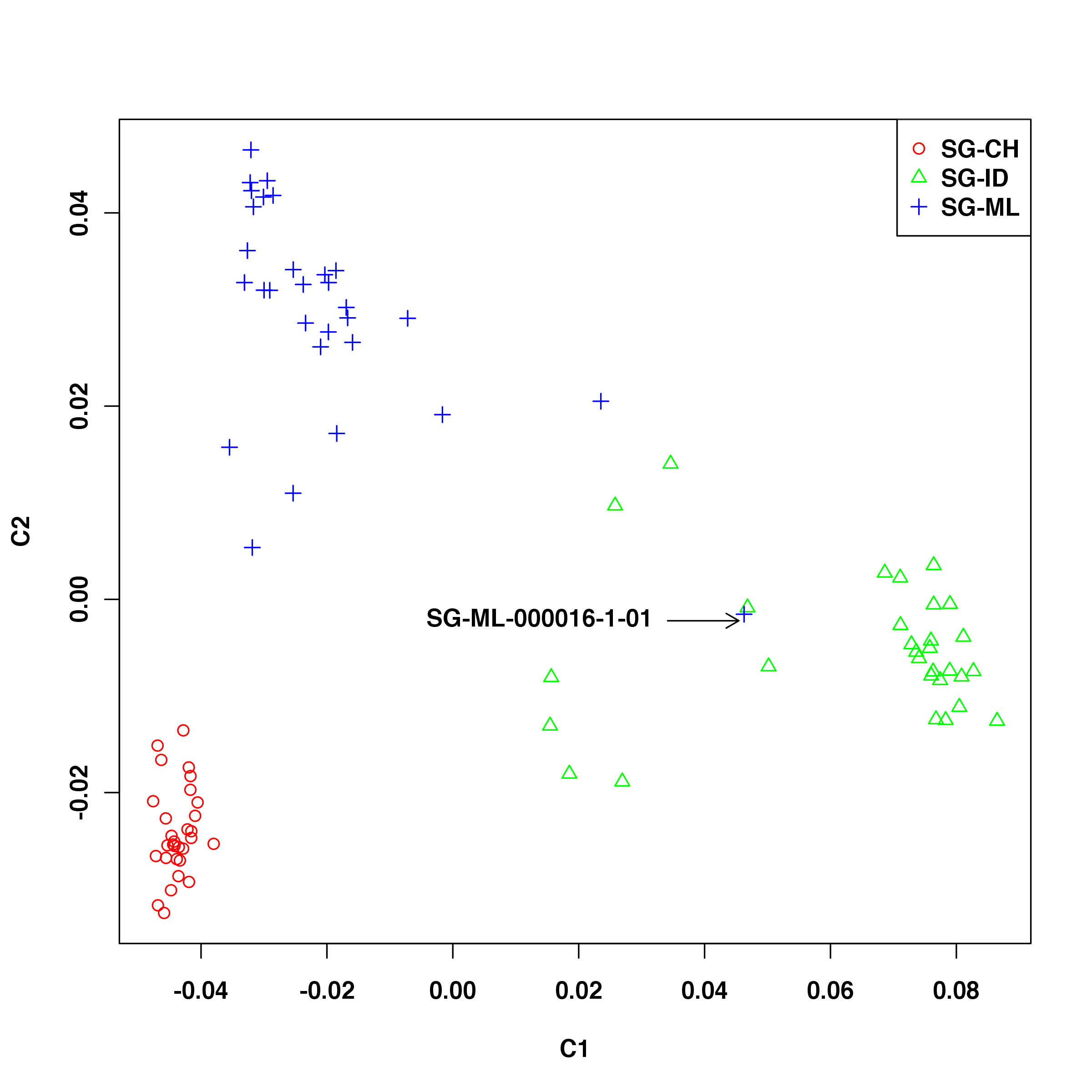

Supplement: Figure S10 — MDS analysis of samples from Singapore. (TIF) [file pone.0029502.s011.tif]

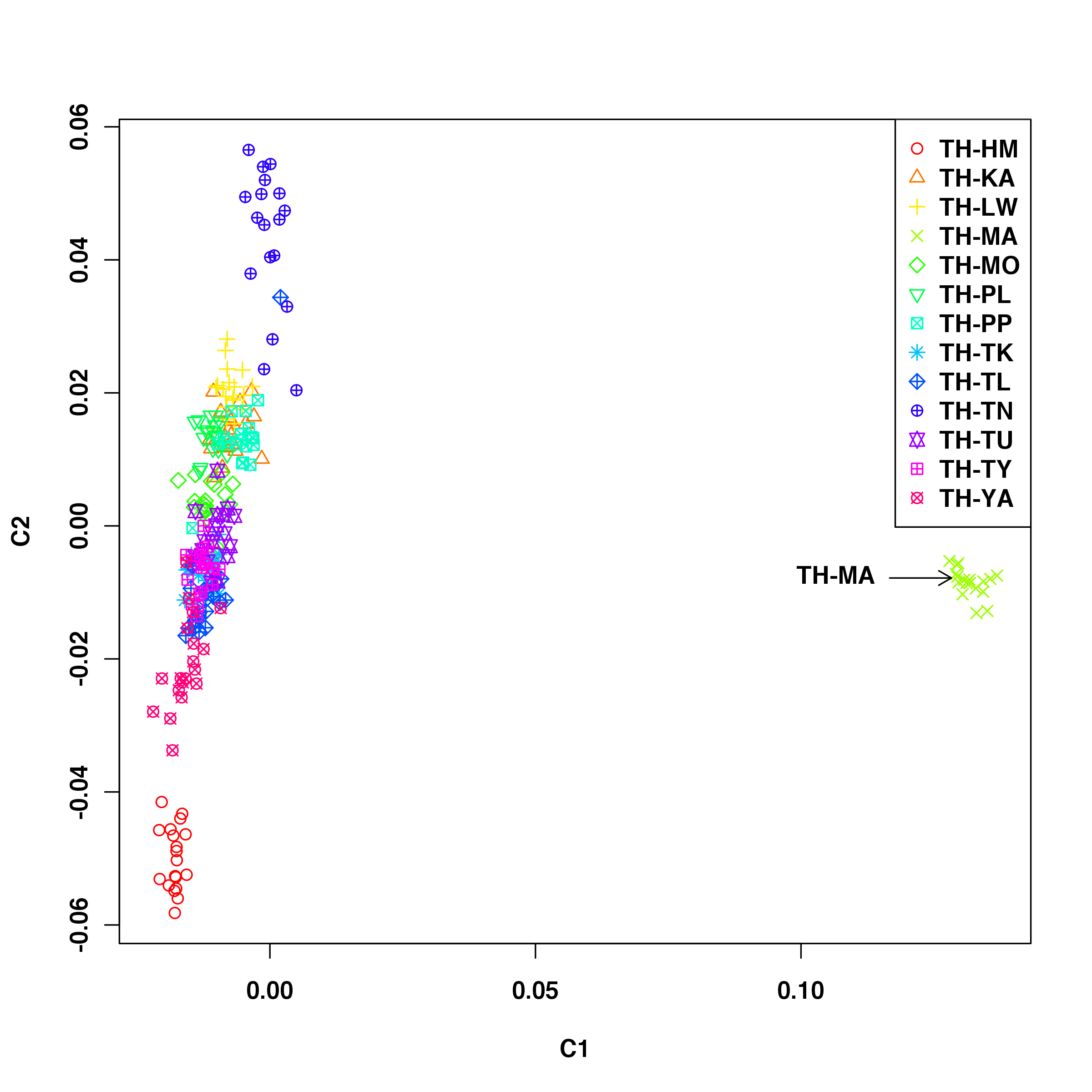

Supplement: Figure S11 — MDS analysis of samples from Thailand. (TIF) [file pone.0029502.s012.tif]

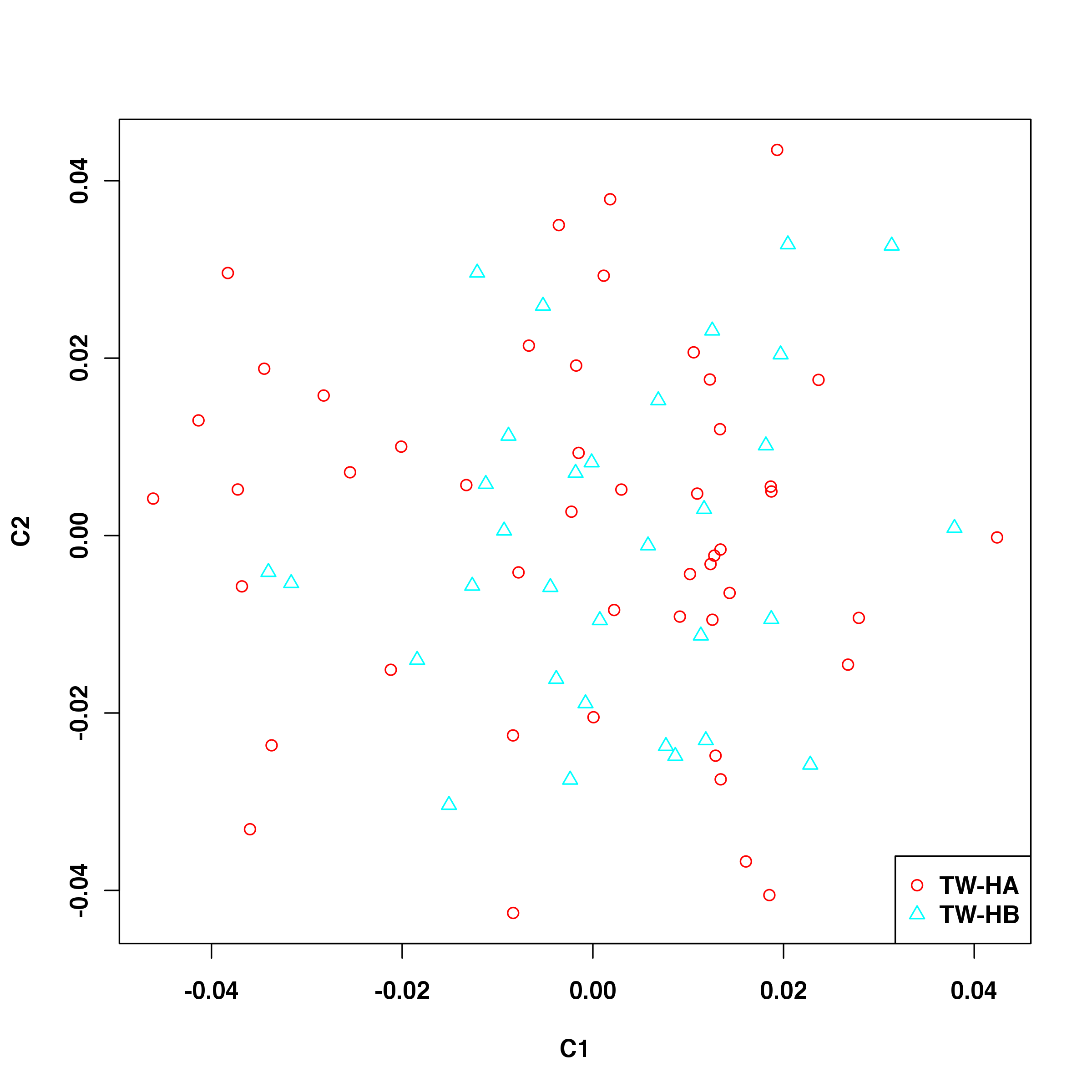

Supplement: Figure S12 — MDS analysis of samples from Taiwan. (TIF) [file pone.0029502.s013.tif]

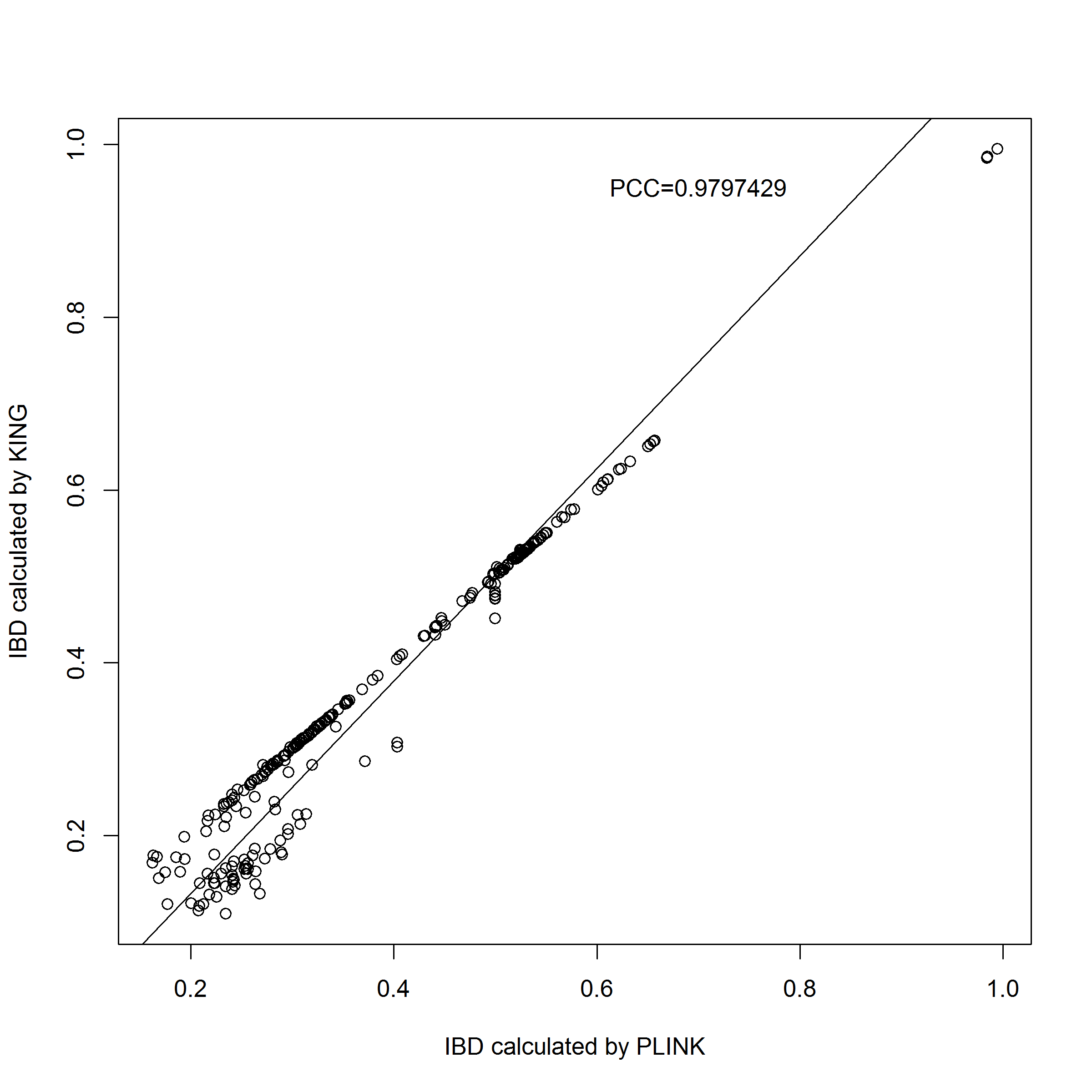

Supplement: Figure S13 — Comparison of IBD calculated by PLINK and that of KING. IBD = P(IBD = 2)+0.5*P(IBD = 1); PCC, Pearson correlation coefficient. (TIF) [file pone.0029502.s014.tif]

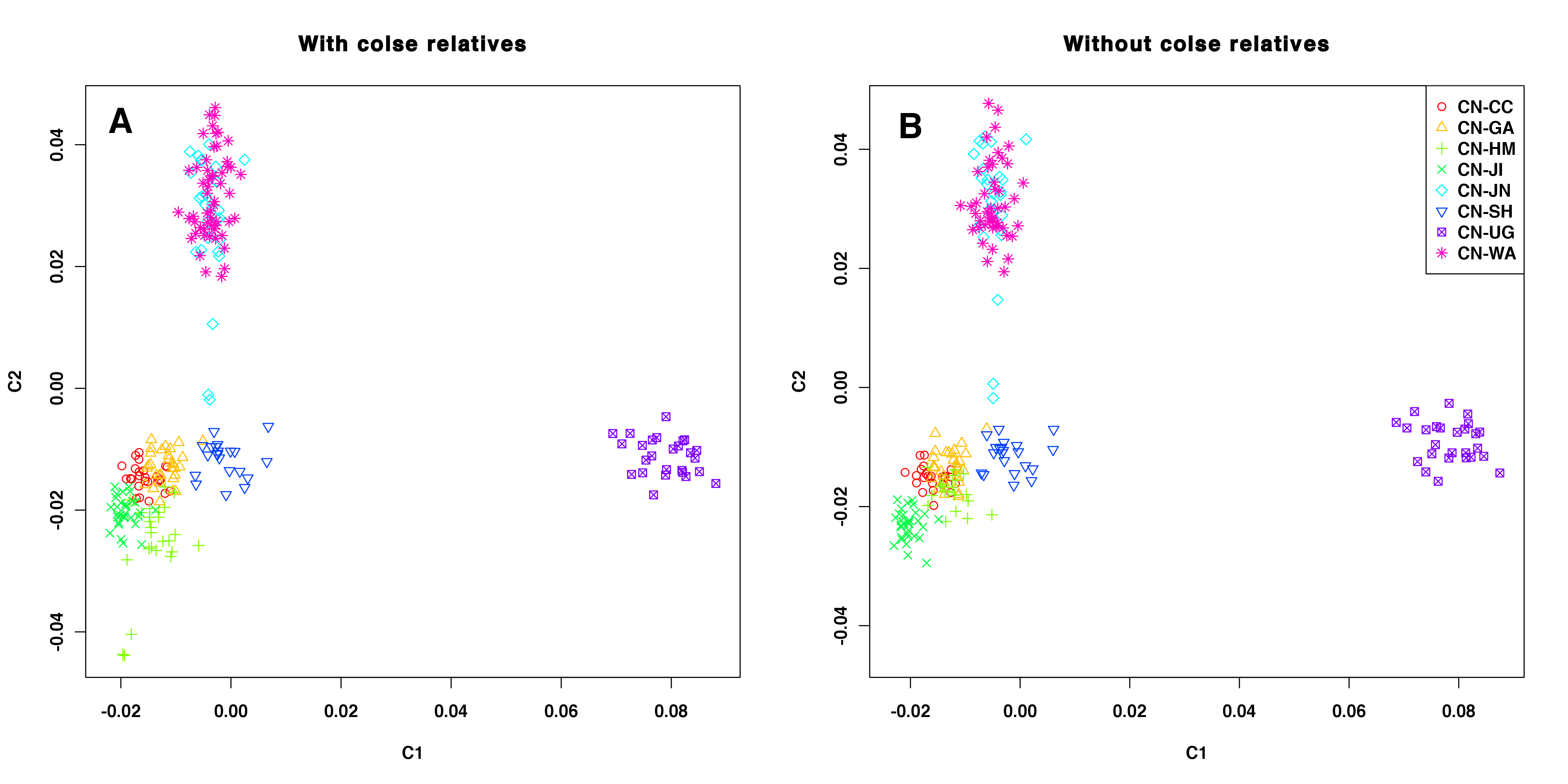

Supplement: Figure S14 — MDS plot of populations from China. (A) Population structure inferred by MDS analysis with close relatives. (B) Population structure inferred by MDS analysis without close relatives. (TIF) [file pone.0029502.s015.tif]

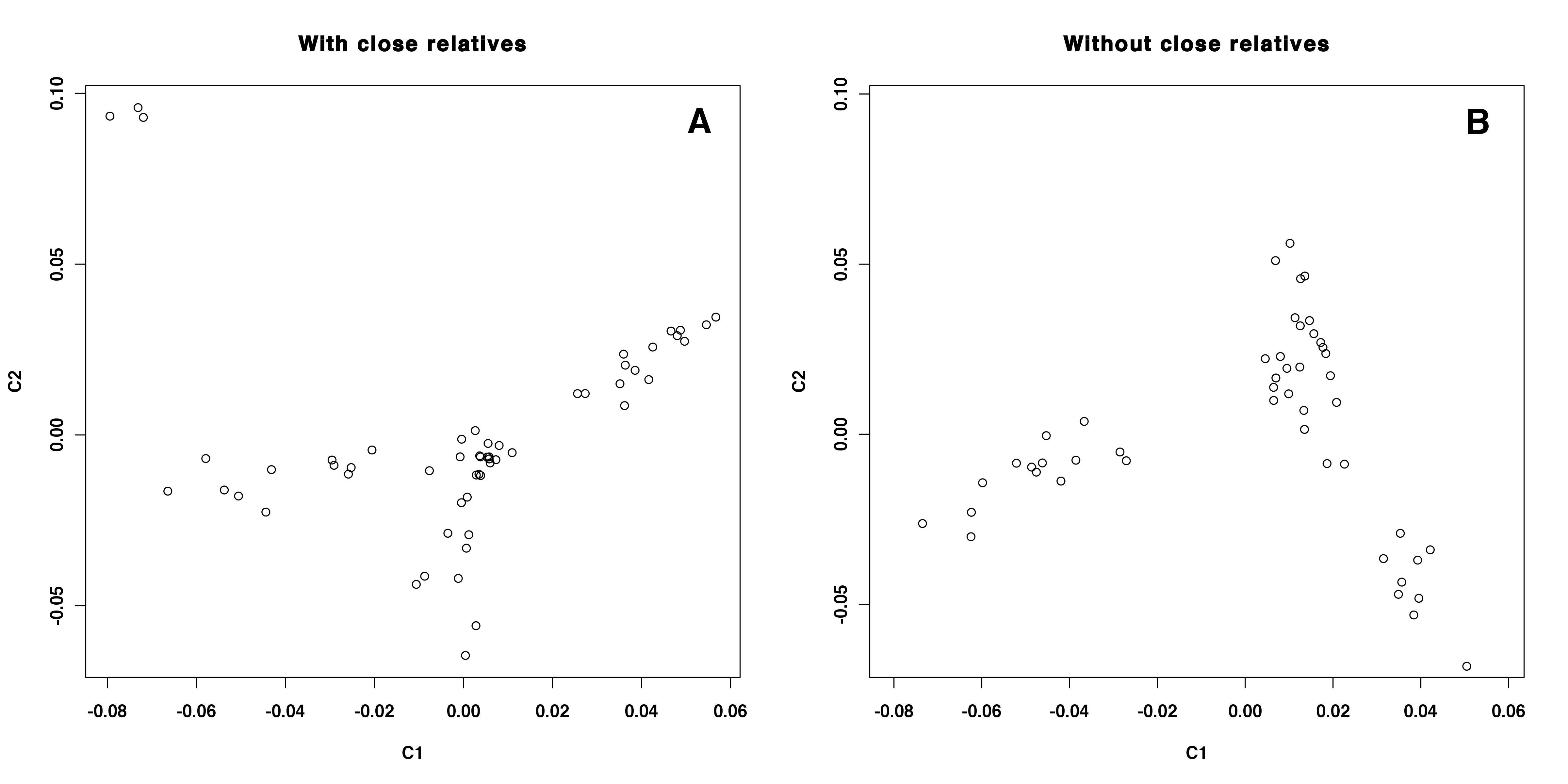

Supplement: Figure S15 — MDS plot of population CN-WA. (A) Population structure inferred by MDS analysis with close relatives. (B) Population structure inferred by MDS analysis without close relatives. (TIF) [file pone.0029502.s016.tif]
